# Electrical signalling beyond diffusion-limited microbial interactions

**DOI:** 10.1093/ismejo/wrag238

**Published:** 2026-09-17

**Authors:** Paulo R F Rocha, Leïla Tirichine

**Affiliations:** Bioelectronics and Bioenergy Research Lab, Centre for Functional Ecology-Science for People & the Planet, Associate Laboratory TERRA, Department of Life Sciences, University of Coimbra, Coimbra 3000-456, Portugal; Nantes Université, CNRS, US2B, UMR 6286, F-44000 Nantes, France; Institute for Marine and Antarctic Studies (IMAS), Ecology and Biodiversity Centre, University of Tasmania, Hobart, TAS 7004, Australia

**Keywords:** bioelectricity, microbial communication, ion-driven signalling, extracellular electron transfer, phycosphere, reaction–diffusion, handover distance

Communication underpins biological organization and functioning across length scales, from microbial consortia to host-associated ecosystems. Ecological interactions have traditionally been interpreted through a chemical description based on diffusible metabolites, quorum-sensing molecules, and receptor-mediated signalling. In this description, interaction range is governed by molecular diffusion, signal uptake or degradation, environmental mixing, source geometry and recipient sensitivity. The phycosphere, the chemically modified region surrounding a phytoplankton cell within which released metabolites and infochemicals can influence neighbouring microorganisms, provides a well-studied example of this local chemical interaction regime (Fig. 1) [1]. For a specified compound, its operational extent is the distance over which the concentration remains sufficiently elevated above background to influence a biological recipient. Under conditions of weak advection, molecular diffusion dominates transport, while uptake, degradation, buffering and dilution restrict signal persistence. If these removal processes are represented by an effective first-order persistence time,${\tau}_s$, the resulting diffusion-loss model has the characteristic length:


(1) \begin{eqnarray*} {\lambda}_s=\sqrt{D_s{\tau}_s} \end{eqnarray*}

**Figure 1:**
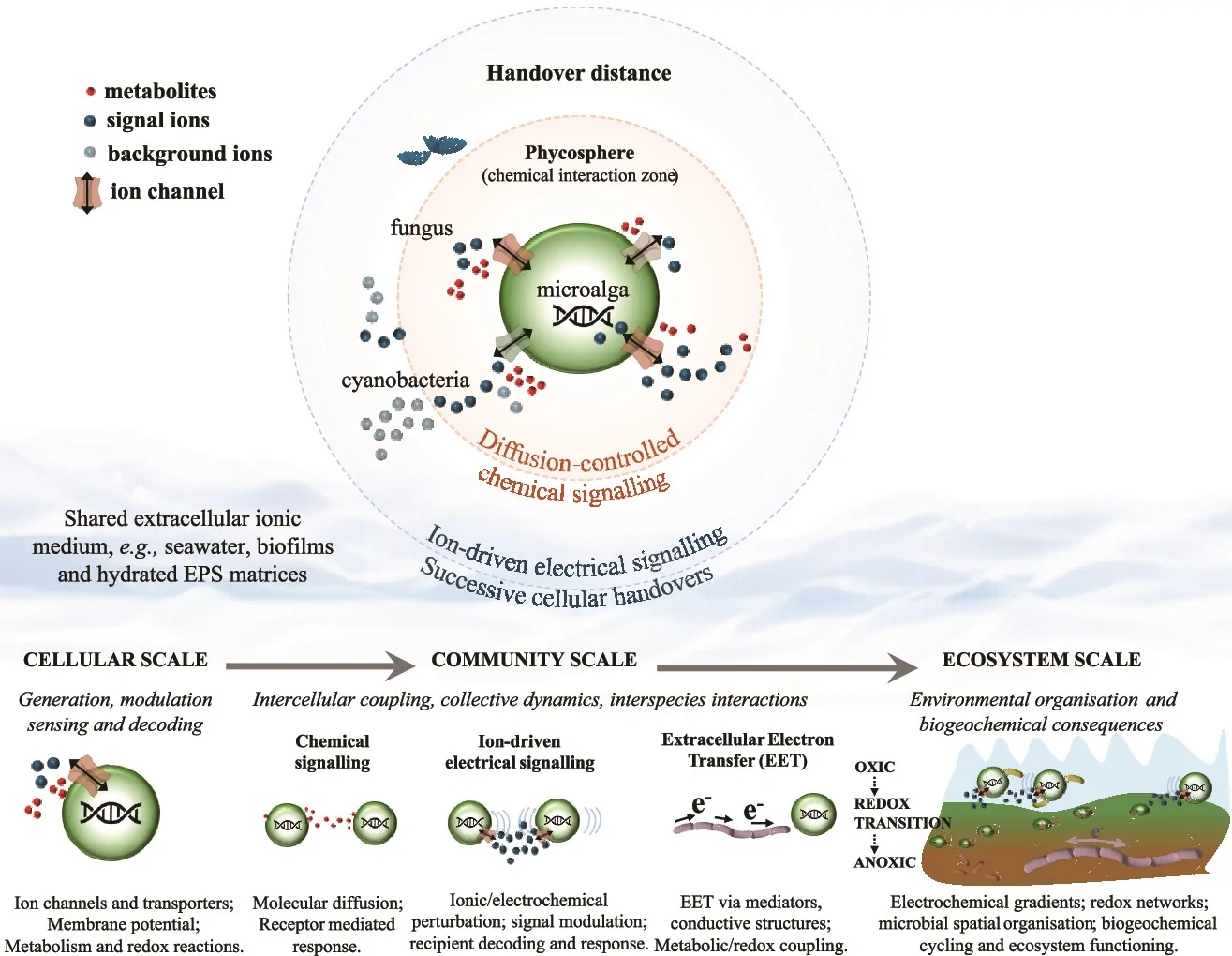
Cross-scale organization of chemical and bioelectrical interactions in microbial ecosystems. The upper panel shows diffusion-controlled chemical interactions within the phycosphere and ion-driven electrical coupling among responsive cells in a shared extracellular medium. The blue region indicates the population-scale handover distance produced through one or more cellular responses. Its extent is schematic and may lie within, overlap with or extend beyond the phycosphere. EET is shown separately as electron transfer through redox-active mediators or conductive biological structures. The lower panels trace chemical signalling, ion-driven electrical signalling and EET from their cellular origins through community-level coupling to ecosystem-level electrochemical gradients, spatial organization and biogeochemical processes. The oxic-anoxic sediment profile illustrates conductor-mediated coupling between spatially separated redox reactions.

Here, ${D}_s$ is the effective diffusion coefficient of signalling species s. The quantity ${\lambda}_s$ has units of length and represents the distance over which an exponentially attenuating perturbation falls to *1/e* of its initial value in a one-dimensional or locally planar profile. Equation 1 therefore provides a reference scale for diffusion-controlled interaction. The actual phycosphere boundary also depends on cell size, source flux, geometry, background concentration, environmental mixing and the concentration or response threshold used to define it. In microfluidic experiments with bacterial communities, fitted spatial fluorescence response profiles yielded communication ranges of 4.7 ± 0.6 μm and 13.4 ± 0.2 μm, for two quorum-sensing systems with irreversible signal uptake, compared with 87.0 ± 6.1 μm for a non-absorbing system [2]. In cytokine diffusion-consumption systems, interaction ranges of ~80–140 μm have been reported [3]. These values show that uptake, source strength, geometry, cellular density, and recipient response thresholds determine the functional diffusion range. Different compounds within the same phycosphere can consequently establish distinct and overlapping chemical interaction zones.

Yet, the phycosphere does not define the outer limit of microbial influence. An extracellular ionic perturbation that reaches a responsive neighbouring cell can be sensed and can induce that cell to alter its own ion fluxes, turning it into a new local source [4]. We refer to this recipient response and regeneration as a cellular handover. Through successive cellular handovers, a physiological state can propagate across a population over distances greater than those traversed by the ions released from the initiating cell. We define handover distance as the maximum separation, within a specified observation period and spatial configuration, over which an initiating ion-driven perturbation elicits a measurable biological response through one or more successive cellular handovers. Its value may lie within, overlap with or extend beyond diffusion-controlled chemical interaction zones, depending on ionic transport, source dynamics, cellular spacing and recipient responsiveness.

For a single passive handover involving ion *i*, the characteristic diffusion-loss length ${\lambda}_i$ is given by Equation 1. For a one-dimensional or locally planar profile, an extracellular perturbation with source amplitude $\Delta{c}_0$ attenuates with distance *x* as $\Delta c(x)=\Delta{c}_0{e}^{-x/{\lambda}_i}$. If $\Delta{c}_{th}$ denotes the recipient response threshold, setting $\Delta c\left({d}_H\right)=\Delta{c}_{th}$ and solving for handover distance ${d}_H$ gives:


(2) \begin{eqnarray*} {d}_H={\lambda}_i\mathit{\ln}\left(\frac{\Delta{c}_0}{\Delta{c}_{th}}\right) \end{eqnarray*}

This expression applies when $\Delta{c}_0>\Delta{c}_{th}$. For a single sender-recipient handover, Equation 2 gives the maximum functional separation at which the attenuated ionic perturbation reaches the recipient response threshold. Thus, ${d}_H$ depends on the diffusion-loss length, source amplitude and recipient sensitivity, whereas ${\lambda}_i$ is the underlying characteristic attenuation length. In a regenerative population, the same local condition applies at each successive handover, allowing the population-scale handover distance to extend beyond the range of the perturbation generated by the initiating cell. Using as a typical example ${D}_{Ca^{2+}}\approx 7.5\times{10}^{-10}\ {m}^2\ {s}^{-1}$ and a characteristic persistence timescale of 80 s gives ${\lambda}_i\approx 2.4\times{10}^{-4}\ m$*,* or ~$250\ \mu m$. We note this value is the characteristic diffusion-loss length, which is distinct from the handover distance itself. Calculation of ${d}_H$ additionally requires the ratio between source amplitude and recipient response threshold. In the idealized exponential profile, a perturbation measured 120 μm from its source would retain ~61% of its initial excess amplitude. The ~120 μm intercellular separation previously analysed in a diatom population therefore lies within one characteristic attenuation length [4]. A recipient at this separation can respond if the remaining perturbation exceeds its response threshold and, if it subsequently generates a new ionic perturbation, the same local condition applies to the next handover. Under idealized complete regeneration, ten successive handovers across comparable intercellular separations would span ~1.2 mm, although the perturbation generated by the initiating cell would only need to reach the first responsive neighbour. This example is illustrative and the realized range depends on source amplitude, recipient threshold, cellular spacing, response time, and the extent of signal regeneration.

Figure 1 summarizes these spatial relationships. It contrasts diffusion-controlled chemical interaction zones with single-step and regenerative ion-driven handovers. It also shows extracellular electron transfer (EET) as a separate process in which electrons move through redox-active mediators or conductive biological structures. EET is discussed separately below and is not included in the handover-distance derivation.

The historical emphasis on chemical communication partly reflects an asymmetry in experimental accessibility. Diffusible metabolites, nutrients and signalling molecules can be chemically identified, quantified and incorporated into established reaction–diffusion descriptions. Microbial electrical activity is more difficult to resolve [5–8]. Electrical signals are often small, heterogeneous and distributed across populations, while conventional electrophysiological approaches were developed mainly for larger excitable cells [8]. Extracellular electrical measurements can also combine contributions from membrane ion fluxes, metabolic activity, redox processes, and the surrounding ionic environment, complicating their biological interpretation. This methodological gap is narrowing through advances in microbial electrophysiology [4, 8], genetically encoded and small-molecule reporters of membrane potential and ion dynamics [9, 10, 14], multielectrode arrays for population-level recordings [7, 11], spatially resolved electrochemical microscopy [12] and bioelectronic interfaces that couple directly to microbial populations [5, 6].

These methods have revealed that ion-driven electrical signalling can couple microbial cells without requiring electron transfer through a biological conductor. In *Bacillus subtilis* biofilms, metabolic stress activates the YugO K^+^ channel and releases intracellular K^+^, producing a propagating extracellular K^+^ wave. The released K^+^ depolarizes neighbouring cells, while feedback between metabolic state and channel gating sustains propagation and coordinates cells across millimetre-scale biofilms [10]. This is a regenerative reaction–diffusion process mediated by extracellular ions and is mechanistically separate from EET and unscreened electrostatic-field propagation. Photosynthetic eukaryotes also exhibit membrane excitability and Ca^2+^-dependent responses [13, 14]. In diatoms, single-domain voltage-gated channels enable rapid Na^+^/Ca^2+^-based action potentials despite the absence of canonical metazoan channel architectures [9]. Collective electrical oscillations have been measured in diatom populations [7], and extracellular ion fluxes can couple neighbouring microalgal cells during acclimation [4]. Ion-channel-associated oscillations have also been reported in *Escherichia coli* biofilms [15], while K^+^ released by *B. subtilis* biofilms can alter the motility of other bacterial species and promote their attraction [16]. These examples intertwine membrane ion transport with intercellular coupling, signal regeneration, and recipient responses, the main cellular processes required for successive handovers.

A second class of microbial electrical process is EET. EET couples intracellular metabolism to extracellular electron donors or acceptors by transferring electrons across the cell envelope and, in some systems, beyond the cell surface. In electroactive microorganisms such as *Geobacter sulfurreducens* and *Shewanella oneidensis*, species-dependent pathways combine multihaem cytochromes with soluble redox shuttles or conductive extracellular structures [17, 18]. These processes differ from ion-driven signalling because the transported charge participates directly in redox reactions and energy metabolism. Cable bacteria provide a distinct example of long-range electron conduction. Conductive periplasmic fibres carry electronic current along multicellular filaments over millimetre-to-centimetre distances [19, 20], coupling sulphide oxidation in deeper anoxic sediment to oxygen or nitrate reduction closer to the sediment surface. The resulting separation of redox half-reactions modifies sediment pH, redox zonation and mineral cycling, thereby reorganizing microbial metabolic niches. EET therefore enables long-range metabolic and redox coupling through defined electron-transfer pathways.

Ion-driven signalling and EET can both couple spatially separated biological activities, although they operate through different charge carriers and pathways [4, 18, 20]. Ion-driven signalling involves dynamic ionic and electrochemical perturbations in a shared extracellular medium. EET transfers electrons via redox-active mediators or along conductive biological structures. These distinctions determine how each process responds to the physicochemical properties of the surrounding environment.

Microbial habitats contain media and structures that support different forms of charge transport. Seawater, porewater, hydrated biofilms, and extracellular polymeric matrices conduct ionic current, while redox-active minerals, conductive biological structures and, in experiments, electrodes can support mediated or electronic charge transfer. Bulk conductivity alone does not determine interaction range, and its influence depends on the transport mechanism, source geometry, temporal dynamics, and recipient responsiveness. Ionic currents and concentration perturbations must also be distinguished from the equilibrium electrostatic potential produced by a localized charge. In seawater, this potential is screened over a Debye length of ~0.3–0.4 nm. This electrostatic screening length is distinct from the micrometre-scale diffusion-loss length in Equation 1 and the functional handover distance in Equation 2. Time-dependent ion gradients and current-associated potential differences can extend across much greater distances because they are maintained by ionic fluxes, sources, sinks, and boundary conditions, even while the bulk solution remains approximately electroneutral. Their dynamics are governed by electrodiffusion, advection, geometry, and biological responses. EET follows a separate physical regime involving redox-mediated electron transfer or electronic conduction along specialized biological structures.

Microbial activity continuously reshapes extracellular electrochemical conditions, but physical coupling alone does not establish communication. We use information-bearing communication only when a biologically generated signal is distinguishable from background fluctuations, can be sensed and decoded by a recipient, and produces a reproducible physiological or behavioural response. Detectability can be quantified using a signal-to-noise measure based on amplitude or frequency-band power relative to matched background measurements, with a predefined detection threshold and false-positive rate. Population-level coherence can improve detectability because correlated cellular contributions accumulate above uncorrelated noise, although spatial averaging reduces single-cell localization. Potentially informative features include amplitude, polarity, duration, frequency, and temporal pattern. Evidence for decoding requires that these features predict recipient responses above chance and that controlled stimulation or replay reproduces the response. Recipient ion channels, membrane properties and downstream pathways determine specificity, whereas the same extracellular perturbation may produce different outcomes in different organisms.

When these criteria are satisfied, ion-driven signalling may coordinate physiological states, resource use, collective behaviour and community stability [4, 10]. Electrical perturbations generated by one species may also influence responsive neighbours through a shared ionic medium, providing a route for interspecies coupling [16]. EET has distinct ecological consequences: coupling spatially separated redox reactions can reorganize electrochemical gradients, mineral transformations, metabolic niches and biogeochemical cycling [17, 19, 20]. EET should be described as metabolic or redox coupling unless a recipient is shown to sense and decode a structured signal.

These predictions can be tested using spatially structured sender-recipient systems. For a single passive handover, sender and recipient cells should be separated by defined distances while ionic composition, bulk conductivity, flow, geometry and confinement are controlled. Independent estimates of ${D}_i$, ${\tau}_i$, $\Delta{c}_0$ and $\Delta{c}_{th}$ would allow ${\lambda}_i$ and the single-step handover distance predicted by Equation 2 to be calculated and compared with the observed recipient response. Regenerative propagation can then be tested by placing one or more responsive cells between the initiating cell and a distant recipient and repeating the experiment after disabling the relevant ion channels or transporters in the intermediate cells. Extension beyond the single-step handover distance only when responsive intermediates are present would provide direct evidence for successive handovers. Simultaneous measurements of extracellular ion concentration, extracellular potential, membrane potential, and intracellular ion dynamics would resolve the sequence from ionic transport to cellular decoding. EET should be examined separately by disrupting redox mediators, cytochromes, or conductive structures and measuring electronic current together with spatially resolved redox chemistry.

At the population scale, multielectrode arrays and low-impedance multidimensional bioelectronic interfaces can be combined with optical reporters of membrane potential, and intracellular ion dynamics to map bioelectrical activity [5–7, 11]. Because extracellular potential recordings integrate ionic, metabolic, and redox contributions, their interpretation should require agreement with ion-specific or spatially resolved redox measurements and causal perturbations. Experiments should quantify attenuation, propagation velocity, response delay, oscillation frequency, synchronization and spatial correlation. Applying defined environmental stimuli or microbial partners would test whether signal amplitude, polarity, duration, frequency, or temporal pattern reliably encodes ecological context. Controlled replay of measured ionic or potential waveforms, with matched electrochemical controls, could determine whether recipients decode these features into distinct biological responses. The resulting interaction ranges, thresholds, delays, and coupling strengths can be incorporated into spatial ecological models to predict effects on community connectivity, synchronization, stability, and spatial organization.

The widespread occurrence of ion channels, membrane potentials and electrochemical gradients suggests deep evolutionary origins for the capacity to generate and respond to electrical perturbations [9, 13]. We propose that their ecological value may extend beyond intracellular homeostasis and that regulated ion fluxes and membrane-potential dynamics could encode physiological state and confer a selective advantage by coordinating responsive neighbours. Information-bearing modulation may therefore be embedded within electrical fluctuations. Comparative studies across bacterial and eukaryotic lineages should identify these signal features and determine when electrophysiological mechanisms support population-level or cross-species communication.

Microbial ecosystems are organized by interacting chemical and electrochemical processes. Diffusion-controlled exchange establishes local interaction zones such as the phycosphere. Ion-driven signalling adds the possibility that responsive cells regenerate extracellular perturbations through successive handovers, allowing a physiological state to propagate beyond the range of the ions released by the initiating cell. EET provides a separate route for coupling spatially separated redox reactions through mediators or conductive structures. Handover distance offers an experimentally testable quantity linking cellular electrophysiology, extracellular transport, cell spacing and recipient response thresholds. Measurements of attenuation, propagation, signal modulation, and decoding can establish when ion-driven coupling carries information, while current and redox measurements can resolve EET-mediated metabolic coupling. Electrical interactions remain governed by physical transport, yet biological regeneration and specialized conductive pathways create ecological length scales that cannot be described by diffusion from a single source alone.

## Data Availability

Data sharing not applicable to this article as no datasets were generated or analysed during the current study.

## References

[1] Seymour JR, Amin SA, Raina J-B et al. Zooming in on the phycosphere: the ecological interface for phytoplankton-bacteria relationships. *Nat Microbiol* 2017;2:17065. 10.1038/nmicrobiol.2017.65 28555622

[2] van Gestel J, Bareia T, Tenennbaum B et al. Short-range quorum sensing controls horizontal gene transfer at micron scale in bacterial communities. *Nat Commun* 2021;12:2324. 10.1038/s41467-021-22649-4 PMC8055654 33875666

[3] Oyler-Yaniv A, Oyler-Yaniv J, Whitlock BM et al. A tunable diffusion-consumption mechanism of cytokine propagation enables plasticity in cell-to-cell communication in the immune system. *Immunity* 2017;46:609–20. 10.1016/j.immuni.2017.03.011 PMC5442880 28389069

[4] Amaral R, Duci D, Cotta FC et al. Ion-driven communication and acclimation strategies in microalgae. *Chem Eng J* 2023;473:144985. 10.1016/j.cej.2023.144985

[5] Cotta FC, Correia D, Amaral R et al. Porous PU/PEDOT:PSS electrodes for probing bioelectricity in *Oscillatoria* sp. cohorts. *Chem Eng J* 2024;498:155480. 10.1016/j.cej.2024.155480

[6] Cotta FC, Amaral R, Bacellar FL et al. A 3D porous electrode for real-time monitoring of microalgal growth and exopolysaccharides yields using electrochemical impedance spectroscopy. *Biosens Bioelectron* 2025;277:117260. 10.1016/j.bios.2025.117260 39983293

[7] Rocha PRF, Silva AD, Godinho L et al. Collective electrical oscillations of a diatom population induced by dark stress. *Sci Rep* 2018;8:5484. 10.1038/s41598-018-23928-9 PMC5883020 29615779

[8] Lo W-C, Krasnopeeva E, Pilizota T. Bacterial electrophysiology. *Annu Rev Biophys* 2024;53:487–510. 10.1146/annurev-biophys-030822-032215 38382113

[9] Helliwell KE, Chrachri A, Koester JA et al. Alternative mechanisms for fast Na^+^/Ca^2+^ signaling in eukaryotes via a novel class of single-domain voltage-gated channels. *Curr Biol* 2019;29:1503–11.e6. 10.1016/j.cub.2019.03.041 PMC6509283 31006567

[10] Prindle A, Liu J, Asally M et al. Ion channels enable electrical communication in bacterial communities. *Nature* 2015;527:59–63. 10.1038/nature15709 PMC4890463 26503040

[11] Masi E, Ciszak M, Santopolo L et al. Electrical spiking in bacterial biofilms. *J R Soc Interface* 2015;12:20141036. 10.1098/rsif.2014.1036 PMC4277093 25392401

[12] Darch SE, Koley D. Quantifying microbial chatter: scanning electrochemical microscopy as a tool to study interactions in biofilms. *Proc R Soc A* 2018;474:20180405. 10.1098/rspa.2018.0405 PMC6304031 30602930

[13] Edel KH, Marchadier E, Brownlee C et al. The evolution of calcium-based signalling in plants. *Curr Biol* 2017;27-79:R667–79. 10.1016/j.cub.2017.05.020 28697370

[14] Vardi A, Formiggini F, Casotti R et al. A stress surveillance system based on calcium and nitric oxide in marine diatoms. *PLoS Biol* 2006;4:e60. 10.1371/journal.pbio.0040060 PMC1370914 16475869

[15] Akabuogu E, da Cunha C, Martorelli V et al. Emergence of ion-channel-mediated electrical oscillations in *Escherichia coli* biofilms. *eLife* 2025;13:RP92525. 10.7554/eLife.92525.3 PMC11928028 40117333

[16] Humphries J, Xiong L, Liu J et al. Species-independent attraction to biofilms through electrical signaling. *Cell* 2017;168:200–9.e12. 10.1016/j.cell.2016.12.014 PMC5497501 28086091

[17] Reguera G, McCarthy KD, Mehta T et al. Extracellular electron transfer via microbial nanowires. *Nature* 2005;435:1098–101. 10.1038/nature03661 15973408

[18] Lovley DR, Holmes DE. Electromicrobiology: the ecophysiology of phylogenetically diverse electroactive microorganisms. *Nat Rev Microbiol* 2022;20:5–19. 10.1038/s41579-021-00597-6 34316046

[19] Pfeffer C, Larsen S, Song J et al. Filamentous bacteria transport electrons over centimetre distances. *Nature* 2012;491:218–21. 10.1038/nature11586 23103872

[20] Meysman FJR, Cornelissen R, Trashin S et al. A highly conductive fibre network enables centimetre-scale electron transport in multicellular cable bacteria. *Nat Commun* 2019;10:4120. 10.1038/s41467-019-12115-7 PMC6739318 31511526

